# High-Grade Leiomyosarcoma Arising in a Previously Replanted Limb

**DOI:** 10.1155/2015/172603

**Published:** 2015-08-23

**Authors:** Tiffany J. Pan, Liron Pantanowitz, Kurt R. Weiss

**Affiliations:** ^1^Department of Orthopaedic Surgery, University of Pittsburgh Medical Center, 3471 Fifth Avenue, Pittsburgh, PA 15213, USA; ^2^Division of Anatomic Pathology, University of Pittsburgh Medical Center, 5230 Centre Avenue, Pittsburgh, PA 15232, USA

## Abstract

Sarcoma development has been associated with genetics, irradiation, viral infections, and immunodeficiency. Reports of sarcomas arising in the setting of prior trauma, as in burn scars or fracture sites, are rare. We report a case of a leiomyosarcoma arising in an arm that had previously been replanted at the level of the elbow joint following traumatic amputation when the patient was eight years old. He presented twenty-four years later with a 10.8 cm mass in the replanted arm located on the volar forearm. The tumor was completely resected and pathology examination showed a high-grade, subfascial spindle cell sarcoma diagnosed as a grade 3 leiomyosarcoma with stage pT2bNxMx. The patient underwent treatment with brachytherapy, reconstruction with a free flap, and subsequently chemotherapy. To the best of our knowledge, this is the first case report of leiomyosarcoma developing in a replanted extremity. Development of leiomyosarcoma in this case could be related to revascularization, scar formation, or chronic injury after replantation. The patient remains healthy without signs of recurrence at three-year follow-up.

## 1. Introduction

Soft tissue leiomyosarcoma is a relatively rare tumor, arising from smooth muscle and comprising approximately 5–10% of all soft tissue sarcomas [1]. Although they typically occur in the uterus, retroperitoneum, intra-abdominal viscera, and blood vessel walls, they can also be found in the bone and soft tissues of the extremities [2].

Few reports exist in the literature of sarcoma occurring in the setting of prior musculoskeletal trauma. Case reports exist in the non-English literature that describe posttraumatic osteosarcoma [3], osteosarcoma arising from posttraumatic myositis ossificans [4], rhabdomyosarcoma after a gunshot wound [5], and sarcoma developing at a fracture site after open reduction and internal fixation [6]. One case report in the English literature regarding rhabdomyosarcoma of the hand following severe trauma was written over half a century ago [7].

We present the case of a patient who sustained a traumatic amputation around the level of the elbow joint as a child and subsequently developed leiomyosarcoma in the replanted limb 24 years later.

## 2. Case Presentation

At the age of 8, the patient was involved in a tractor accident, and his right arm was severed just distal to the elbow joint. He underwent multiple surgical procedures, resulting in successful replantation of the limb. Of note, immunosuppression was not required given that he had undergone a replantation and not a transplant. Despite suboptimal function, he was working full time in construction at the time of presentation.

**Figure 1:**
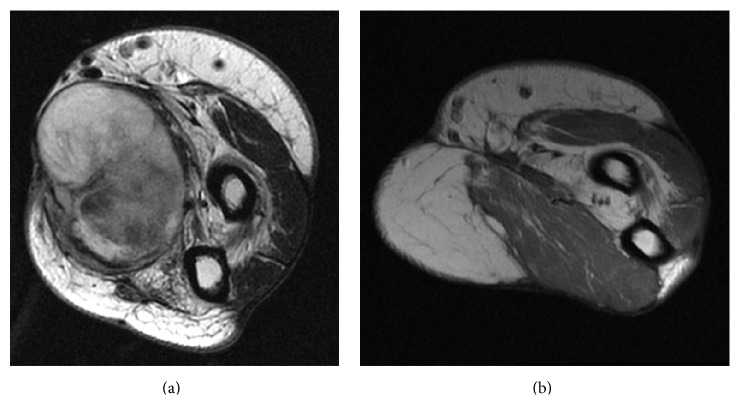
Preoperative T2 axial MRI image showing a heterogenous, subfascial mass (a) compared with postoperative T1 axial MRI image showing no evidence of residual mass (b).

24 years subsequent to the accident, the patient returned to the hand surgeon who had performed his replantation as a child when he noticed a mass approximately the size of an orange in his right volar medial forearm. MRI was obtained that demonstrated 10 × 10 × 10 cm heterogenous, subfascial mass, ostensibly originating from the area of the interosseous membrane (Figure 1(a)). His surgeon performed an incomplete excision. Pathology showed a high-grade leiomyosarcoma with 20% necrosis and no angiolymphatic invasion.

He was subsequently referred to our tertiary sarcoma center for evaluation by a musculoskeletal oncologist. At that time, he had essentially normal neurologic hand function with a composite grasp and an elbow ankylosed at approximately 30° of flexion. The arm had enumerable scars with evidence of multiple prior soft tissue transfers, but no draining sinuses or evidence of infection. The most recent incision had been made along a previous incision line and extended proximally through the antecubital fossa. A large mass was palpable in this region. Repeat imaging studies 6 weeks after the incomplete excision revealed that the lesion had essentially completely regrown to its preoperative size and shape.

A lengthy discussion was held with the patient, whose primary concern was to salvage the limb if at all possible. Due largely to his extreme physical and emotional investments to save the arm as a child, he was unwilling to accept the possibility of an amputation. An angiogram was performed, identifying the ulnar artery as the only major vessel feeding the distal arm, though there was an extensive collateral tree. It was determined at this time that excision with negative margins would be technically challenging but feasible.

The patient was taken for surgical resection with a limb-sparing procedure and brachytherapy. The median nerve was adhered to the tumor, but the musculoskeletal oncologist was able to salvage it by sacrificing some of the branches. The ulnar artery, however, was encased by tumor. Vascular surgery evaluated the patient intraoperatively and was able to identify a Doppler signal from the brachial artery proximally but could not find a good vessel for a bypass. Therefore, the decision was made to ligate the ulnar artery despite it being the predominant vascular supply to the distal limb. Surprisingly, the radial pulse was dopplerable and the hand remained warm and soft. The tumor was removed en bloc and brachytherapy catheters were placed under a vacuum-assisted-closure (VAC) dressing as described [8].

**Figure 2:**
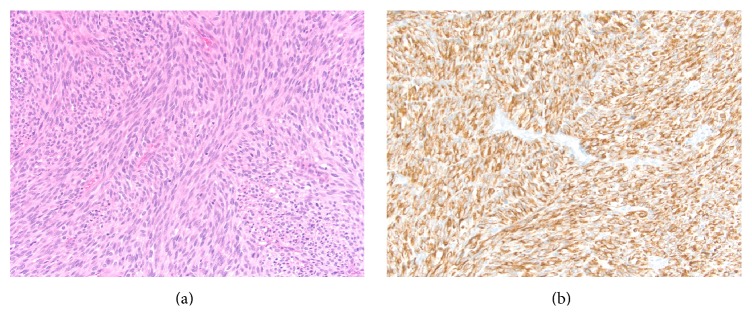
Pathology from the time of definitive surgery revealed a high-grade spindle cell sarcoma growing in fascicles that invaded subfascial tissue ((a), H&E). Immunohistochemical stains demonstrated strong, diffuse positivity for desmin ((b), immunostain). A grade 3 leiomyosarcoma was diagnosed with stage pT2bNxMx.

Postoperatively, the radial pulse remained dopplerable, and the hand remained well perfused. Pathology revealed a 10.8 cm residual high-grade leiomyosarcoma with 5–10% necrosis and 28 mitoses per 10 high-powered fields (Figure 2). Margins were microscopically negative. He underwent brachytherapy and the catheters were removed on postoperative day 8 along with replacement of new VAC dressing. Three days later, he underwent free flap reconstruction of the forearm with split thickness skin grafting and delayed primary closure of the remainder of the wound. The ulnar nerve was transferred to motor branches enervating segmental portions of the flap.

**Figure 3:**
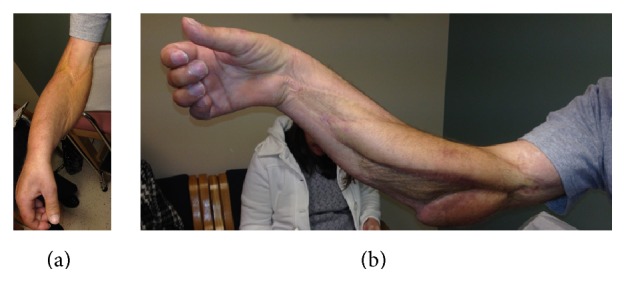
At most recent follow-up, the patient is sensate in the median and radial nerve distributions with weak wrist and finger flexion and extension.

After recovering from these operations, the patient had 4 cycles of ifosfamide and doxorubicin adjuvant chemotherapy. At his most recent follow-up, three years postoperatively, the patient had no evidence of local or systemic recurrence. He was back to work full time as a laborer and his wounds were completely healed (Figures 1(b) and 3).

## 3. Discussion

Significant trauma has been reported to be a rare risk factor for development of neoplasia, particularly in the case of burns. A review of 80 years of burn literature found 412 case reports of neoplasm arising in burn scars. While squamous cell carcinoma was by far the most common at 71%, 5% of the cases were sarcoma, including one report of leiomyosarcoma [9]. The mean latency interval between time of burn injury and time of tumor diagnosis was 31 years. Healing by secondary intention, nonhealing wounds, and fragile scars were identified as significant risk factors in the development of burn scar neoplasms “Marjolin's ulcer”. One review of 11 burn scars over a period of 20 years found a predilection for burn scar carcinomas to arise in the flexion crease of limbs, thought to be a result of repetitive microtrauma from otherwise low impact everyday activities [10].

The pathogenesis of burn scar malignancies is not precisely understood. Initially, it was hypothesized that the damaged tissue released toxins that induced cellular mutations [11]. Another theory regarding the development of skin cancer after trauma is displacement of live epithelial cells into the deep tissue with a concurrent inability of the damaged tissue to regulate against the invading cells [12]. The current prevailing theory is that scar tissue forms an immunologically privileged site due to damage to lymphatic systems that hinders natural immunosurveillance, allowing neoplastic changes to occur [9, 13]. However, there is little scientific data available in the literature to support these postulates. In small series of patients, p53 has been found to be absent or dysfunctional in scar neoplasms, but no large scale scientific studies have been conducted that demonstrate a common pathophysiology for development of burn scar carcinomas [14, 15].

Leiomyosarcoma is typically an intra-abdominal or retroperitoneal tumor, though, in a study that spanned 7 years, 12% were located in the extremities and all but one was subfascial. While a vascular origin was suggested by the relationship of the tumor to the vessels in the majority of cases, definitive diagnosis could only be established in 5 of the cases. Tumor size >10 cm at presentation, mitotic rate >19 per 10 HPF, marginal or intralesional excision, and lack of adjuvant radiotherapy were found to be significant risk factors for progression of leiomyosarcomas located in the extremities. In their series, 2-year and 5-year disease-free survival rates were 42.3% and 32.6%, respectively [1].

Another series of 66 patients over 22 years with leiomyosarcoma arising from the soft tissues of the extremities found that only 16% were located in the upper extremities. Mortality in this series was similar with 50% survival at a mean of 3 years after diagnosis. Identified risk factors for mortality included tumor size >5 cm^3^, MTS stage III, presence of metastases, and axial anatomic location. Radiation and chemotherapy did not appear to directly affect outcome, but patients who underwent surgery with adjuvant therapy trended towards longer survival compared with those treated with surgery alone [2].

To our knowledge, this is the first case reported in the literature of sarcoma development in a replanted limb and joins a very small number of cases of posttraumatic sarcoma in the extremities. The development of leiomyosarcoma in the upper extremity is unusual and even more unique in the setting of a previously replanted arm. While the precise etiology of posttraumatic sarcoma development has not been well established, analogous progression of burn scars into malignancies is proposed to result from development of an immune privileged site that hinders the body's ability to recognize and eliminate cancerous cells. This explanation is also plausible in the setting of severe trauma such as an amputation with multiple procedures required for successful replantation due to disruption of local lymphatic systems in both situations, which prevent tumor specific antigens from being carried to regional lymph nodes and allow for relatively unrestricted tumor growth [13, 16].

In this case, similar to the average latency period seen in burn scar neoplasms, the patient developed a mass 24 years after his initial trauma. Although he presented with several poor prognostic indicators including a tumor size of >10 cm and a high number of mitoses per HPF, he was aggressively treated with wide resection and brachytherapy followed by chemotherapy in accordance with his determination to salvage the limb, minimize the chance of local recurrence, and eradicate micrometastatic disease. He has maintained baseline function of his replanted arm and remains free of local recurrence and evidence of metastases at three-year follow-up.
